# Our Professors and Their Qualifications

**Published:** 1866-10

**Authors:** 


### Miscellaneous.

### Our Professors and their Qualifications.

(From the New York Medical Record.)

The very responsible and honorable position occupied by medical teachers would seem to entitle them to a freedom from criticism, but that they cannot claim such an immunity is apparent in the efforts which have been made to remedy the evils connected with their calling. A great number of our professors are men who, by their profound learning, skillful practice, and extensive experience, are justly entitled to our esteem and confidence. But the proportion is much too small compared with those who are totally unqualified for the positions which they occupy. The truth of this admission is as patent as the existence of such a state of things is mortifying. There is, in fact, not a medical school in the land that would not be benefited by a thorough sifting of its faculty, and too many would be in need of a sieve that would be coarse enough to allow the majority of the members to drop easily through.

It is easy enough, however, to prove that many teachers are not what they should be; but when we look about us for a practical plan for at once ridding the professional corps of these useless and harmful members, it becomes altogether another matter. Here, indeed, is the principal difficulty of the subject, and it does not grow any the less by the aid of any light which discussion can throw upon it.

There is no number to the suggestions which have been offered in reference to insuring a competency on the part of the professors of the schools, but they have all proved to be merely visionary when subjected to the rigid test of practicability. The reason for this is made obvious enough when we examine into the manner in which our teachers are appointed, and the claims which they have upon the different colleges for their positions. Our schools, not being under the patronage of the government, are of necessity strictly private enterprises. A number of gentlemen conclude that a certain locality is a fit one for a college, and all that remains to be done in order to act upon such a conclusion is the obtaining of a charter. This latter is accomplished generally without much trouble. The State, when it has given power to the faculty of granting, under certain expressed conditions, a diploma of doctor of medicine, does not trouble itself with reference to details. As it does not furnish any pecuniary means, the expenses of the institution must be borne by the parties who are interested, and these are most generally the teachers themselves. These are mainly self-appointed, and, of course, contribute their quota of the necessary funds. It is, after all, on their part, a purely business arrangement; a certain amount of capital is invested, and a given return is expected. If the income of the college, from the attendance of its students, is found sufficient to pay a decent percentage on the principal, we have what is usually called "a successful school."

When we consider the danger of having, under such circumstances, professors who have more money than brains, we have reasons for congratulating ourselves that our good teachers are after all in the majority; that while we have here and there an objectionable member in a faculty, we are not so badly off as we might be. This may be, in part, accounted for in this way:—Each faculty knows that, in order to gain the confidence of his patrons, and the consequent success of the institution, it must have decently qualified men to teach the different branches, and this is acknowledged to be such a necessity that brains must be represented as well as capital. The evil, then, which might otherwise be expected from the system of self-constitution, carry with themselves, to a certain extent, their own antidote.

Beyond the remedy which we here have for incompetency of our teachers, we can hardly hope to go, unless we have a centralized power in our government which shall control all appointments, and settle all questions of qualification. But there is no such centralized power in our government; and even if it did exist, its exercise would by no means be productive of those gratifying results which we see set forth in the monarchies of Europe. Our rulers, contrary to those of other countries, represent in the main the lower classes; and we have, with shame be it said, men in our high offices who are as innocent of the charge of being educated as they are of being respectable. If, then, we were to centralize the power of such officials, we should have even less surety than at present of either good teachers or respectable schools. There is, fortunately for us, no power in government which can compel the appointment of any candidate to a professorial chair in any college, or exact from such aspirant any stated qualifications. It is infinitely better for the cause of medical education that our different colleges should have such matters in their own hands. The educated portion of the community—the real patrons of such institutions—are to be the judges of the fitness of the professors for their respective positions, and they are the ones who must exact from them a compliance with their requirements. It is in this very education of the people that the safety of all our institutions for learning lies, and just in proportion as the standard is raised will our system of teaching be perfect. Arguing upon general principles, if the faculty, as a whole, is a poor one, the college suffers; and if a very indifferent and unqualified one, the school is ruined. A faculty, for its own protection and perpetuation, must of necessity have good men, and these must be in the majority. We are glad that these bodies appreciate the importance of securing the best obtainable talent, and it is a source of gratification that their efforts have been, in a measure, crowned with success. We are, however, far from being satisfied that it is as well as it should be. There are still too many objectionable members in almost every faculty, who are poor teachers, bad lecturers, and hobbyists, and who, for the good of the respective institutions, must be weeded out. But by exercising our patience, and allowing time to bring about a demand for better men, the desired result will be effected. The current of public opinion is steadily setting in the right direction, and we have good reason to hope that at no very distant date we may be able to claim as able, as distinguished, and as experienced teachers as can anywhere be found.

